# Barriers to Implementing Registered Nurse–Driven Clinical Decision Support for Antibiotic Stewardship: Retrospective Case Study

**DOI:** 10.2196/54996

**Published:** 2024-05-23

**Authors:** Elizabeth R Stevens, Lynn Xu, JaeEun Kwon, Sumaiya Tasneem, Natalie Henning, Dawn Feldthouse, Eun Ji Kim, Rachel Hess, Katherine L Dauber-Decker, Paul D Smith, Wendy Halm, Pranisha Gautam-Goyal, David A Feldstein, Devin M Mann

**Affiliations:** 1 Department of Population Health New York University Grossman School of Medicine New York, NY United States; 2 Northwell New Hyde Park, NY United States; 3 Department of Population Health Sciences University of Utah Salt Lake City, UT United States; 4 Department of Internal Medicine University of Utah Salt Lake City, UT United States; 5 Department of Family Medicine and Community Health University of Wisconsin School of Medicine and Public Health Madison, WI United States; 6 Department of Medicine University of Wisconsin School of Medicine and Public Health Madison, WI United States; 7 University of Wisconsin-Madison School of Nursing Madison, WI United States; 8 Department of Medicine New York University Langone New York, NY United States

**Keywords:** integrated clinical prediction rules, EHR, electronic health record, implementation, barriers, acute respiratory infections, antibiotics, CDS, clinical decision support, decision support, antibiotic, prescribe, prescription, acute respiratory infection, barrier, effectiveness, registered nurse, RN, RN-driven intervention, personnel availability, workflow variability, infrastructure, infrastructures, law, laws, policy, policies, clinical-care setting, clinical setting, electronic health records, RN-driven, antibiotic stewardship, retrospective analysis, Consolidated Framework for Implementation Research, CFIR, CDS-based intervention, urgent care, New York, chart review, interview, interviews, staff change, staff changes, RN shortage, RN shortages, turnover, health system, nurse, nurses, researcher, researchers

## Abstract

**Background:**

Up to 50% of antibiotic prescriptions for upper respiratory infections (URIs) are inappropriate. Clinical decision support (CDS) systems to mitigate unnecessary antibiotic prescriptions have been implemented into electronic health records, but their use by providers has been limited.

**Objective:**

As a delegation protocol, we adapted a validated electronic health record–integrated clinical prediction rule (iCPR) CDS-based intervention for registered nurses (RNs), consisting of triage to identify patients with low-acuity URI followed by CDS-guided RN visits. It was implemented in February 2022 as a randomized controlled stepped-wedge trial in 43 primary and urgent care practices within 4 academic health systems in New York, Wisconsin, and Utah. While issues were pragmatically addressed as they arose, a systematic assessment of the barriers to implementation is needed to better understand and address these barriers.

**Methods:**

We performed a retrospective case study, collecting quantitative and qualitative data regarding clinical workflows and triage-template use from expert interviews, study surveys, routine check-ins with practice personnel, and chart reviews over the first year of implementation of the iCPR intervention. Guided by the updated CFIR (Consolidated Framework for Implementation Research), we characterized the initial barriers to implementing a URI iCPR intervention for RNs in ambulatory care. CFIR constructs were coded as missing, neutral, weak, or strong implementation factors.

**Results:**

Barriers were identified within all implementation domains. The strongest barriers were found in the outer setting, with those factors trickling down to impact the inner setting. Local conditions driven by COVID-19 served as one of the strongest barriers, impacting attitudes among practice staff and ultimately contributing to a work infrastructure characterized by staff changes, RN shortages and turnover, and competing responsibilities. Policies and laws regarding scope of practice of RNs varied by state and institutional application of those laws, with some allowing more clinical autonomy for RNs. This necessitated different study procedures at each study site to meet practice requirements, increasing innovation complexity. Similarly, institutional policies led to varying levels of compatibility with existing triage, rooming, and documentation workflows. These workflow conflicts were compounded by limited available resources, as well as an implementation climate of optional participation, few participation incentives, and thus low relative priority compared to other clinical duties.

**Conclusions:**

Both between and within health care systems, significant variability existed in workflows for patient intake and triage. Even in a relatively straightforward clinical workflow, workflow and cultural differences appreciably impacted intervention adoption. Takeaways from this study can be applied to other RN delegation protocol implementations of new and innovative CDS tools within existing workflows to support integration and improve uptake. When implementing a system-wide clinical care intervention, considerations must be made for variability in culture and workflows at the state, health system, practice, and individual levels.

**Trial Registration:**

ClinicalTrials.gov NCT04255303; https://clinicaltrials.gov/ct2/show/NCT04255303

## Introduction

Antibiotic resistance is a major public health risk, with more than 35,000 deaths each year in the United States due to antibiotic-resistant bacterial infections [1,2]. Overprescribing and misuse of antibiotics for upper respiratory infections (URIs) remain the most significant combined factors causing antibiotic resistance [3,4]. In the United States, up to 50% of all outpatient antibiotic prescriptions for URIs are inappropriate [5,6].

An estimated 80%-90% of antibiotic prescribing occurs in outpatient settings, such as doctors’ offices, urgent care facilities, and emergency departments [7-9]. From 1996 to 2010, 72% of adult patients in primary care with a diagnosis of acute bronchitis received antibiotics contrary to guideline recommendations against antibiotic treatment, and prescription rates actually increased during this time frame [10]. Patients with sore throats received antibiotics 61% of the time when the prevalence of group A streptococcus, the only clear indication for antibiotics, was only 10% in adults [11].

By providing real-time evidence-based data to assist providers (physicians, nurse practitioners, and physician assistants) in estimating the likelihood of a patient having either pneumococcal pneumonia or group A streptococcus, electronic health record (EHR)–integrated clinical prediction rules (iCPRs) can help address prescriber-level barriers to antibiotic stewardship and reduce antibiotic prescribing for URIs in primary care [12-15]. Indeed, CPRs have already been validated to successfully distinguish between viral and bacterial respiratory infections [16-18].

While potentially effective, there is low uptake of the iCPR tools among physicians in primary care practices, thus indicating implementation barriers to antibiotic stewardship iCPRs among physicians [19]. This outcome is consistent with other literature, indicating that physicians perceive antibiotic stewardship as onerous and would require substantial assistance to change their antibiotic prescribing behaviors [20]. Due to these limitations associated with the physician-driven iCPR implementation model, such as “alert fatigue” and time constraints [21,22], the iCPR intervention was adapted so antibiotic stewardship tasks could be delegated to other qualified members of the medical team.

A registered nurse (RN)–driven implementation model of iCPR for low-acuity URIs has the potential to be an effective alternative to the physician-driven implementation model. RNs have demonstrated equivalent symptom resolution compared to physicians when using protocols to improve ambulatory care across a number of chronic diseases [23] as well as the treatment of acute minor illnesses [24,25]. Therefore, the iCPR intervention was adapted for RNs to include the identification of patients with low-acuity URI followed by clinical decision support (CDS)–guided RN visits. The intervention was implemented in February 2022 as a stepped-wedge trial in primary and urgent care practices within 4 academic health systems in New York, Wisconsin, and Utah [26].

Despite a seemingly straightforward URI clinical workflow, the RN-driven iCPR intervention encountered significant barriers early on during implementation. While these issues were pragmatically addressed as they arose during study implementation, a systematic assessment of the barriers to implementation is needed to better understand and address these barriers. The CFIR (Consolidated Framework for Implementation Research) [27] has been widely used to guide the systematic assessment of multilevel implementation contexts to identify contextual determinants of implementation success [28]. Using the updated CFIR as a guide [29], we sought to identify and categorize the barriers experienced during the implementation of the RN-driven antibiotic stewardship “iCPR3” intervention.

## Methods

### Overview

We performed a retrospective case study, collecting quantitative and qualitative data from expert interviews, study surveys, routine check-ins with practice personnel, and chart reviews over the first year of implementation of the iCPR3 intervention. We used the updated CFIR [29] to characterize the initial barriers to adapting a URI iCPR intervention for RNs in ambulatory care.

### Ethical Considerations

The study protocol and procedures were approved by the NYU Langone Health institutional review board, which served as the study’s single institutional review board (NYULH Study: i19-01222). Informed consent was received from all participants. Documentation of consent was waived for this study. All study data reported in this manuscript are deidentified. Compensation was not provided for participation.

### Study Intervention

**Figure 1:**
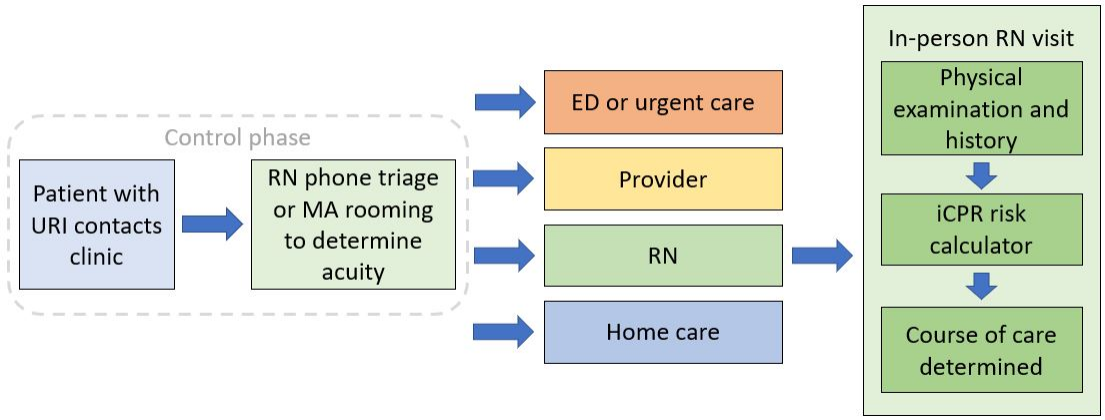
iCPR3 intervention URI patient workflow including patient contact, triage, and nurse visit. "Provider: includes physicians, NPs, and physician assistants. ED: emergency department; MA: medical assistant; NP: nurse practitioner; RN: registered nurse; URI: upper respiratory infection.

The study intervention consists of triage followed by an in-person iCPR–guided RN visit for patients with low-acuity URIs (Figure 1). RNs perform telephone triage (or in urgent care, an RN or medical assistant performs a similar assessment through a rooming protocol) for patients reporting cough or sore throat symptoms to assess acuity, need for primary care, urgent care, or ED visit, and appropriateness for an in-person iCPR–guided RN visit. In the urgent care setting, the assessment is dichotomous as either a need for a provider visit or appropriateness for an iCPR-guided RN visit. The triage tool consists of a prepopulated note template integrated into the EHR system designed to document patient symptoms and their severity and determine the most appropriate level of care. Triage algorithms were based on institutional triage resources for decisions about ED or urgent care visits, primary care visits, and home care [30].

Patients triaged as low acuity and appropriate for an RN visit are invited for an in-person RN visit that replaces the standard of care provider visit. During the RN visit, guided by iCPR tools, the RN evaluates a patient to determine their risk of bacterial infections of strep pharyngitis (sore throat) or pneumonia (cough). Prepopulated note template EHR tools lead RNs through a focused history and physical examination. Once an RN completes the patient history and physical examination, they use an iCPR tool specific to cough or sore throat to calculate the risk of bacterial infection based on the patient’s vitals, symptoms, and pertinent history [26]. The iCPR tools are informed by the CPRs [17,18,31] used in the iCPR1 and iCPR2 studies [12,19], which were validated in prior studies among patients with acute respiratory illnesses [17,18,31]. The CPRs are integrated into the EHR, and upon completion of the calculator, the level of risk with an approximate probability of having either strep pharyngitis or pneumonia is displayed. After completion of the risk calculator, the RN is linked by the EHR to an order set specific to the level of risk, along with relevant patient education.

### Setting and Participants

The iCPR3 intervention study was implemented in February 2022, as a randomized controlled stepped-wedge trial, in 43 primary and urgent care practices associated with 4 academic medical centers including 2 in New York, 1 in Wisconsin, and 1 in Utah. To be eligible for participation, a practice must include general internal medicine, family medicine, or urgent care practices. Furthermore, practices must have at least 1 RN full-time equivalent capable of performing triage within the EHR and in-person RN visits.

For this case study, purposive sampling was used to select experts with key knowledge and insight on study implementation from members of the research team and study practices. This sample included research study team members engaged in the implementation of the iCPR3 study (ie, research coordinators, research assistants, and investigators) and study practice personnel (ie, RNs, RN or practice managers, and providers) from each study site (academic medical center). At least 2 research study team members per site participated in semistructured interviews, with those experts determining which practice personnel to include in their data collection. All RNs participating in the iCPR3 intervention were included in study acceptability surveys and routine implementation check-ins with study staff.

Of note, approximately 8 months into iCPR3 implementation, 1 New York–based study site withdrew from the intervention study due to limited practice recruitment and insurmountable barriers to implementing the intervention. Interviews were still performed with site personnel and their comments are included in these analyses.

### Data Collection

#### Interviews

A semistructured interview guide containing questions based on the 5 domains of CFIR [27,29] was developed. The CFIR constructs supported the research team in defining topics for the interviews and ensured that all major domains in the framework that influence implementation were addressed. Interview questions did not explicitly name or ask participants to name the CFIR domains or constructs. The interviews were performed via in-depth email interviews [32], in which research study team interviewees were asked questions to identify which of the 48 CFIR constructs were perceived as current barriers to iCPR3 implementation and provided detailed descriptions of the identified barriers and strategies that have already been used by the iCPR3 research study team.

#### Surveys and Routine Check-Ins

The perspectives of practice personnel were incorporated into the case study based on notes from surveys; individual interviews; or written feedback from RNs, providers, and RN and practice managers collected over the implementation period as routine intervention study procedures. As this was a pragmatic study, study staff routinely elicited informal feedback from practice personnel throughout intervention implementation to identify barriers and improve intervention implementation.

At 6 and 12 months post-RN visit implementation, participating RNs completed a short survey that asked about burnout, job satisfaction, and comfort levels with performing tasks related to treating patients reporting cough and sore throat. The survey also collected information on ease of use of the EHR tools as well as feedback on elements of the intervention, such as training, and recommendations.

#### Chart Review

Clinical workflows and EHR note templates (triage and RN visits) use in the first 12 months of implementation were collected via chart review. A subset of EHR template uses initiated was evaluated for appropriateness and completeness. To determine the total number of potential patients in a practice eligible for triage template use, patients with visits resulting in a diagnosis code for cough or sore throat were documented (*International Classification of Diseases-10* [*ICD-10*] codes: R05, R07.0, J20.9, J06.9, and J18.9). The EHR records related to the visit were reviewed to determine patient eligibility for triage and document the workflow leading to the patient visit (ie, how the appointment was scheduled, by whom, and whether appointment notes were present).

#### CFIR Domains and Constructs

The CFIR was used to retrospectively describe the implementation process of the iCPR3 intervention to identify determinants in this process. Only the determinants relevant to the iCPR3 intervention implementation process were described. The CFIR is composed of 48 constructs sorted into 5 major domains including innovation, outer setting, inner setting, individuals, and implementation process [27,29]. Operationalization of CFIR domains for this study are shown in Table 1.

**Table 1:** Operationalization of Consolidated Framework for Implementation Research domains.

Domain		Description
Innovation		iCPR3^a^ tools and protocol
Outer setting		Health system and state policies/climate
Inner setting		Participating practices within the health systems
**Individuals**		
	Roles	RNs^b^, RN/practice managers, medical directors, providers, administrative staff, and patients
	Innovation deliverer	RNs
	Innovation recipients	Patients
	Implementation facilitators	Practice personnel who contributed to the success of study implementation
Implementation process		How the iCPR3 intervention was implemented

^a^iCPR3: integrated clinical prediction rule 3.

^b^RN: registered nurse.

### Data Coding and Analysis

Insights gathered from the surveys, chart reviews, and formal and informal check-ins with study practice personnel helped inform research study team members’ responses to the semistructured interview guide. The written responses and notes collected from the email interviews were analyzed using techniques of qualitative content analysis, inspired by a deductive-directed approach, deemed applicable because the data were analyzed in light of an existing framework [33]. The analysis was performed by 3 authors (ERS, LX, and JK) in a stepwise interactive process. The first step in the analysis, after reading all transcripts, notes, and written responses to obtain an understanding of the whole, was to develop initial coding nodes and subnodes based on the domains and constructs of the CFIR [29].

In the second step, units of analysis, such as sentences or sections of thought, were deductively coded into the nodes and subnodes. Third, the coded text was rated based on the recommended method described by the authors of CFIR, Damschroder and Lowery [34]. In the rating process, a consensus process was used to assign a rating to each construct obtained from each study site. The ratings reflected the positive or negative influence and the strength of each construct that emerged based on the coded text. When all constructs obtained from all study sites were rated, we compared and compiled ratings for each construct across study sites. Constructs were coded as missing, not distinguishing between positive or negative implementation factors (0), or weakly (+1/–1), or strongly (+2/–2) distinguishing low from high implementation factors (Table 2).

**Table 2:** Coding criteria used to assign ratings to CFIR^a^ constructs for the iCPR3^b^ implementation.

Rating	Criteria
–2	The construct is a strong negative influence impeding implementation efforts. The majority of respondents describe explicit examples of how a construct manifests itself in a strongly negative way.
–1	The construct is a negative influence impeding implementation efforts. Respondents make general statements about the construct manifesting in a negative way with or without concrete examples and there is sufficient information to make an indirect inference about the generally negative influence. This rating can indicate a weak negative effect or that there is a mixed effect of different aspects of the construct but with a general overall negative effect.
0	A construct has neutral influence if (1) it appears to have a neutral effect (purely descriptive) or is only mentioned generically without valence, (2) there is no evidence of positive or negative influence, (3) respondents contradict each other, or (4) there are positive and negative influences that balance each other out, the construct has some positive influence whereas other influences are negative and, overall, the effect is neutral.
+1	The construct is a positive influence in facilitating implementation efforts. Respondents make general statements about the construct manifesting in a positive way with or without concrete examples and there is sufficient information to make an indirect inference about the generally positive influence. This rating can indicate a weak positive effect or that there is a mixed effect of different aspects of the construct but with a general overall positive effect.
+2	The construct is a positive influence in facilitating implementation efforts. The majority of respondents describe explicit examples of how a construct manifests itself in a strongly positive way.
Missing	Respondents were not asked about the presence or influence of the construct or, if they were asked about a construct, their responses did not correspond to the intended construct and were instead coded to another construct.

^a^CFIR: Consolidated Framework for Implementation Research.

^b^iCPR3: integrated clinical prediction rule intervention.

## Results

### Overview

Barriers and facilitators to implementation were identified within the CFIR domains and constructs and are presented within the frame of CFIR domains including innovation, outer and inner settings, individuals, and implementation process (Table 3).

**Table 3:** CFIR^a^ implementation construct ratings by the iCPR3^b^ study site based on the rating criteria.

CFIR domains and constructs^c,d^			Construct influence^e^				
			New York A	New York B	Utah	Wisconsin	Overall
**Innovation**							
	B. Innovation evidence base		+2	0	0	0	0
	D. Innovation adaptability		–1	–2	+1	–1	–1
	F. Innovation complexity		–2	Missing	–1	–1	–1
**Outer setting**							
	B. Local attitudes		Missing	–1	–1	Missing	–1
	C. Local conditions		–2	–2	–2	–2	–2
	D. Partnerships and connections		+1	0	0	0	0
	E. Policies and laws		–2	–2	0	+2	–2
	**G. External** **p** **ressure**						
		2. Market pressure	Missing	0	Missing	0	0
		3. Performance measurement pressure	–1	Missing	Missing	Missing	–1
**Inner setting**							
	**A.** **S** **tructural** **c** **haracteristics**						
		1. Physical infrastructure	–1	–1	Missing	0	–1
		2. IT infrastructure	0	–2	Missing	0	0
		3. Work infrastructure	–2	–2	–2	–1	–2
	B. Relational connections		Missing	–1	–2	+1	–1
	C. Communications		–1	Missing	–2	+2	–1
	**D. Culture**						
		2. Recipient centeredness	Missing	–1	Missing	Missing	–1
		3. Deliverer centeredness	–2	–1	–2	+1	–1
		4. Learning centeredness	0	–1	Missing	+1	0 (mix)
	E. Tension for change		–1	–1	–1	+1	–1
	F. Compatibility		–1	–2	–2	+2	–2
	G. Relative priority		–2	–2	–2	–1	–2
	H. Incentive systems		–1	0	–2	+1	–2
	I. Mission alignment		0	0	+1	+1	0
	**J. Available** **r** **esources**		+2	Missing	+1	0	+1
		2. Space	0	–1	Missing	0	0
		3. Materials and Equipment	0	Missing	0	–1	0
	K. Access to knowledge and information		+2	+1	–1	+1	+1
**Individuals**							
	**Roles subdomain**						
		A. High-level leaders	+1	–1	0	+2	0 (mix)
		B. Mid-level leaders	0	–1	0	+1	0 (mix)
		C. Opinion leaders	0	Missing	–2	Missing	–2
		D. Implementation facilitators	Missing	Missing	0	+1	+1
		E. Implementation leads	Missing	Missing	Missing	+1	+1
		F. Implementation team members	Missing	Missing	Missing	+1	+1
		G. Other implementation support	Missing	Missing	Missing	Missing	Missing
		H. Innovation deliverers	–1	–1	0	+1	–1
		I. Innovation recipients	–1	–1	–2	+1	–1
	**Characteristics subdomain**						
		B. Capability	–1	–1	0	+1	–1
		C. Opportunity	–2	–2	–1	0	–2
		D. Motivation	–1	0	–2	+1	–1
**Implementation process**							
	A. Teaming		+1	0	+2	+1	+1
	**B. Assessing** **n** **eeds**						
		1. Innovation deliverers	+2	–1	+1	+2	+2
		2. Innovation recipients	0	Missing	Missing	Missing	0
	C. Assessing context		+2	–1	+1	+2	+2
	D. Planning		+2	0	0	0	0
	E. Tailoring strategies		+2	0	0	+1	0
	**F. Engaging**		+2	0	–1	+2	+2
		1. Innovation deliverers	+2	–2	+1	+2	+2
		2. Innovation recipients	0	Missing	0	0	0
	G. Doing		+1	–2	–1	Missing	–1
	**H. Reflecting and evaluating**						
		1. Implementation	+2	0	+1	+1	+1
		2. Innovation	Missing	Missing	Missing	+1	+1
	I. Adapting		+1	–1	–1	+1	+1

^a^CFIR: Consolidated Framework for Implementation Research.

^b^iCPR3: integrated clinical prediction rule intervention.

^c^Construct lettering and numbers correspond with Damschroder et al [27].

^d^Only constructs applicable to the iCPR implementation are cited.

^e^–2: strong negative influence; –1: weak negative influence; 0: neutral influence; 0 (mix): mixed positive and negative influences, which balanced each other; +1: weak positive influence; +2: strong positive influence; missing: not asked or miscoded.

### Outer Setting

*Local conditions*, primarily driven by the COVID-19 pandemic, served as one of the strongest barriers to implementation as COVID-19 impacted nearly every aspect of implementation from changes in workflows and staffing availability to patient volume and URI care protocols. There were observed changes to URI care protocols including shifts from in-office care to telehealth and redirection to urgent care, driven by COVID-19–testing requirements and hesitancy from both patients and practices to have on-site care. Furthermore, COVID-19 affected *local attitudes* among practice staff as health issues and burnout led to staff shortages, turnover, and shifting of responsibilities. These barriers were further compounded by regional nursing shortages and financial incentives that drew RNs out of primary care practices.

Policies and laws, such as state regulatory laws and institutional policies, also had a strong impact on the study procedures and implementation. RN scope-of-practice varied by state and between institutions. Wisconsin has existing RN delegation protocols, allowing for more clinical autonomy among RNs than at institutions in New York and Utah. This required additional training and modification to the RN visit portion of the intervention at institutions, where RNs had a more limited scope of practice and could not function autonomously. For example, the New York sites were required to adopt a “co-visit” structure to ensure that providers could oversee RN visits. This created additional scheduling constraints and complexity, as well as an unanticipated burden for providers. Multimedia Appendix 1 shows the analysis of performance measurement pressure and the innovation construct.

### Inner Setting

Within the construct of *structural characteristics*, *work infrastructure* served as a strong barrier to the intervention implementation as practices across institutions experienced staff changes, RN shortages and turnover, and competing responsibilities that all hindered their ability to effectively participate in the study. Notably, at practices with only 1 RN, implementation was negatively impacted as clinic participation was dependent on 1 individual, whereas at other practices, study responsibilities were distributed across multiple RNs. Within the *culture* construct, a norm of limited *deliverer centeredness*, related to the prioritization of the needs and desires of RNs, served as a barrier to the implementation of this RN-focused intervention. As patient (*recipient centeredness*) and provider preferences were prioritized over RN activities, the innovation activities that would have been performed by the RNs were overridden. For example, to ensure patient autonomy, if a patient preferred to see a provider, they were not scheduled for an RN visit even if they were eligible. Similarly, at most institutions (except those with more RN autonomy), RNs tended to defer to providers in terms of preference and final decisions. Therefore, if the provider preferred seeing a patient themselves, the patient, even if eligible for an RN visit, would not be seen by an RN.

Overall, *relational connections*, specifically the RN-provider dynamic, negatively affected implementation. RNs in the study did not always have open bidirectional communication with providers, thus limiting the self-efficacy of RNs to explain or justify intervention-related activities. As observed within the *culture* construct, many practices had limited *deliverer centeredness*, typically deferring to providers to make final decisions, and therefore RNs were hesitant to push these boundaries or make decisions that were contrary to a provider’s preferences. In particular, some sites mentioned some practices having poor relationships among practice staff, even requiring team-building training in some instances. On the other hand, this was less of a barrier at practices, where RNs had more clinical autonomy or had developed stronger relationships within the practice.

Communications culture within practices served as a barrier to effectively implementing aspects of the study; for example, some practices did not have a culture of communicating with patients prior to visits in the form of triage or lacked formalized documentation as information was often conveyed informally (eg, verbal, secure chat message, and free-text note). In some practices, a strong communication system between RNs (ie, a chat channel used by most RNs) served as a facilitator to innovation implementation by allowing RNs to support and answer each other’s questions.

The intervention’s *compatibility*, or lack thereof, with existing workflows was a strong barrier to implementation, as the necessary intervention-specific workflow adaptions required great effort on the part of the practice if not already in place (eg, front desk forwarding eligible patients for triage, RNs performing triage after appointments had been scheduled, and filling out EHR note templates as opposed to free text). As the new study workflow required changes to the status quo, *tension for change* also served as a barrier since practices perceived little anticipated benefit from the study as compared to the difficulty of change. Relatedly, *relative priority* of the intervention was a strong barrier as competing clinical responsibilities and the voluntary nature of the study meant staff would not prioritize study-related tasks.

Overall, there was a lack of *incentive systems* in place related to study activities, which hindered RN participation. While gift card incentives for RNs performing triage were used, these tended to incentivize the same RNs already using the tools as opposed to encouraging new RNs to participate. Additionally, at institutions where RNs were unable to bill for visits and did not receive any other recognition for their efforts, this lack of incentives was a strong barrier to participation. One institution was able to reduce the influence of this barrier by providing incentives to RNs through continuing education credits, an employee recognition fund, and paid time for training.

Multimedia Appendix 1 presents the analyses of physical infrastructure, IT infrastructure, access to knowledge and information, available resources, learning-centeredness, and mission alignment.

### Individuals: Characteristics Subdomain

Both *capability* and *motivation* were barriers to implementation. As these tools were new to many of the participating RNs, they were less confident in their skills and required continuous feedback, training, and support. In addition, RNs were not motivated to participate in the study largely due to competing priorities, lack of a strong incentive, and COVID-19–related stress and burnout. *Opportunity* was also a strong barrier, as RNs did not have many opportunities to use the innovation tools. Conflicting responsibilities, staff shortages, workflow barriers, patient volume, and patient eligibility were observed as contributors to this barrier.

Multimedia Appendix 1 shows the analyses of roles subdomain constructs high-level leaders, mid-level leaders, opinion leaders, innovation deliverers, innovation recipients, implementation facilitators, implementation leaders, and implementation team members. Multimedia Appendix 1 shows the analyses of the implementation process domain constructs assessing context and assessing needs, innovation deliverers, doing, planning and tailoring strategies, teaming, engaging the innovation deliverers, reflecting and evaluating, and adapting.

## Discussion

### Principal Findings

This case study identified numerous barriers to the successful implementation of iCPR3, an RN-driven antibiotic stewardship intervention. Many of the identified barriers are consistent with those observed in other interventions that sought to alter nursing responsibilities and workflows within primary care [35,36]. The most impactful barriers were noted within the outer setting, and these conditions were observed to influence the inner setting constructs. The effects of COVID-19 served as an overarching barrier that impacted nearly all implementation constructs, shifting the culture and conditions at many participating practices as well as decreasing the capacity of practices to engage in activities perceived as nonessential. These barriers, however, were less prevalent within clinics that had previously established workflows with patient care within the RN role description. Takeaways from this study can be applied to support integration and improve uptake during the implementation of other RN delegation protocols involving CDS tools into existing workflows.

Policies impacting innovation deliverers’ (RNs) clinical autonomy at both the state and institutional levels need to be considered when developing RN delegation protocols as they can impact implementation depending on compatibility with existing workflows. As a multisite study with implementation spanning 3 states, the differing state regulatory laws and institutional policies dictating RN scope-of-practice had a substantial impact on the compatibility of the iCPR3 implementation at each site. This was evident in the higher rate of RN visits occurring in practices in Wisconsin compared to New York. At the Wisconsin study site, there were established delegation protocols for RNs to see patients with minimal provider supervision. In contrast, for the 2 New York study sites, a more complex “covisit” design was developed, which involved joint scheduling of the iCPR3 RN visit followed immediately by a visit for the provider to see the patient and confirm the RN plan of care. The addition of a provider visit component increased the intervention’s dependency on already limited provider availability, thus inhibiting the ability to schedule the iCPR3 RN visits even when a patient was appropriate and willing and an RN was available to conduct the visit. As observed in other RN delegation protocols, considerations for local regulations must be made when assessing the viability of implementing these types of interventions [36].

Consideration of practice-level culture and work infrastructure is also essential for the successful implementation of an intervention that includes RN delegation protocols. This implementation study revealed impactful differences in existing workflow expectations that affected RN capability and intervention complexity. One unexpected barrier was the influence of practice personnel who were part of the local workflow but were not directly involved in the implementation of the iCPR tools. For example, at one institution, successful implementation of the intervention was reliant on administrative staff to forward patients reporting cough and sore throat to participating RNs for triage. Implementation planning with greater efforts to clarify practice-level workflows, identifying potential stakeholders early on, and engaging these personnel who ultimately support the innovation deliverers can support a successful implementation.

Similarly, when delegating provider tasks to RNs, it is important to secure provider buy-in early on in the implementation process, even with a seemingly RN-focused intervention. Consistent with previous research demonstrating the importance of RN-provider relationships in job satisfaction [37], this study showed that power dynamics between providers and RNs can serve as a barrier to RN intervention engagement. With a culture of deference to providers, many RNs did not want to overstep these boundaries and would not engage with the intervention if there was any perceived resistance from practice providers. Barriers experienced due to this power structure were further compounded when poor relations existed between RNs and providers. Furthermore, as seen in other clinical academic partnerships, future implementation efforts would benefit from more active engagement of leadership at all levels [38].

Future clinical delegation interventions may also need to consider alternate care mechanisms to account for unexpected shifts in clinic workflows. Due to the timing of the implementation, one of the largest observed barriers to implementation was the COVID-19 pandemic, which amplified nearly all other barriers and created additional unique challenges. As an intervention specifically designed for in-person care, the shift toward telemedicine driven by the pandemic [39] had a particularly negative impact on implementation. One study institution piloted a program to divert all patients with URI to telemedicine visits with a centrally employed nurse practitioner, which bypassed all potential points of intervention for the iCPR study. Further diverting potentially eligible patients away from primary care practices was the increased popularity of urgent care centers [40], which served as an expedient solution for patients with URI seeking to avoid long wait times at many primary care practices. Incorporating alternate care mechanisms to provide agility in the intervention may support the success of study implementation. Similarly, integrating CDS tools with existing EHR tools and templates can help minimize changes in workflow, thereby allowing interventions to be resilient in the face of unforeseen events.

As observed in this case study, the pandemic also directly impacted practice staff and their ability to participate in activities beyond the essential, including research. Practices across all study institutions experienced nursing staff shortages due to RNs themselves being sick, covering for others who are sick, or leaving the practice altogether, thus resulting in a redistribution of responsibilities. An increased workload, along with outside stressors, led to increased reported stress and burnout among practice staff [41], making it difficult for them to view the study as a daily priority. The voluntary nature of the study and these conflicting responsibilities greatly reduced the opportunity for RNs to use the innovation and participate fully. This was particularly evident in practices that required greater workflow modifications. Practices with existing expectations of note documentation and template use facilitated implementation; however, in other practices, the lack of RN familiarity with these EHR functions required the creation of additional training and workflow modification efforts, as well as a greater perceived effort burden on the part of RNs.

Future implementation should consider the value of face-to-face communication in encouraging engagement and team building during the implementation process [42]. In addition to its impact at the institutional and practice level, the effects of COVID-19 hindered the implementation process itself, especially early in the planning phase by limiting in-person interactions and creating communication barriers [43]. With nearly all communication occurring remotely, interactions to collect practice workflow information and engage stakeholders were perceived as less efficient, requiring additional follow-up meetings and hindering the development of relationships of the study team with leadership and innovation deliverers. When in-person practice visits by the research team became feasible, an improvement in practice responsiveness and innovation uptake was observed [42].

This study had several limitations. First, the use of an emailed in-depth interview hindered the study team’s ability to probe respondents for further information at the moment, potentially limiting the collection of further details that may have impacted the interpretation of interview responses. However, the emailed format increased the feasibility of conducting a long interview and created an opportunity for study sites to compile perspectives from multiple team members, thus improving the richness of information provided. Second, the reported barriers and facilitators were self-reported and not directly observed and are therefore based on the perceptions of the study site research teams. Similarly, as the data collection was primarily retrospective, it may be subject to recall bias. We attempted to mitigate this by conducting semistructured interviews during the implementation process. Finally, this analysis was performed prior to the completion of implementation at all sites and analysis of the primary intervention effectiveness outcomes. Therefore, it was not possible to link perceived implementation constructs to intervention outcome measures, and additional implementation construct influences may have been missed.

### Conclusions

Both between and within health care systems, significant variability exists in workflows for patient intake and triage. Even in a relatively straightforward clinical workflow, seemingly nuanced workflow and culture differences appreciably impacted successful intervention adoption. Barriers to intervention adoption existed within multiple constructs and domains. When implementing a system-wide clinical care intervention, stakeholders should consider the variability in workflow policy and culture at the health system, practice, and individual levels, as well as create accommodations for changing care patterns.

## Appendix

Multimedia Appendix 1 Consolidated Framework for Implementation Research domain barrier analysis results.

## Abbreviations

CDS: clinical decision support
CFIR: Consolidated Framework for Implementation Research
EHR: electronic health record
ICD-10: International Classification of Diseases-10
iCPR: integrated clinical prediction rule
RN: registered nurse
URI: upper respiratory infection

## Data Availability

The data sets used and analyzed during this study contain personally identifiable information and are therefore not made publicly available. Data are available from the corresponding author upon reasonable request.
