# Supplementary material for: Barriers to Implementing Registered Nurse–Driven Clinical Decision Support for Antibiotic Stewardship: Retrospective Case Study
Source: JMIR Form Res. 2024 May 23;8:e54996. doi: 10.2196/54996 (PMC11157178; doi:10.2196/54996)
Supplement: Multimedia Appendix 1 [file formative_v8i1e54996_app1.docx]

## Appendix

## CFIR Domain Barrier Analysis Results

### Innovation

The lack of *Innovation Adaptability,* particularly within EHR (Epic vs. Touchworks) constraints, and perceived *Innovation Complexity*, due to its multi-step nature and use of EHR functionality not frequently employed by study RNs or practice providers, served as contributing factors that affected our ability to implement all feedback collected during the planning stage of implementation. This barrier, however, was minor with some adaptations made to the tool, but changes were primarily made to adapt trainings to fit the local context or needs, and unanticipated trainings and communications were conducted throughout the implementation period to address RN and provider questions.

### Outer Setting

The absence of *Performance Measurement Pressure* in the form of performance quality metrics aligned with the innovation procedures had a slightly negative impact on implementation by diminishing the perception of the innovation as a priority and not incentivizing RNs to participate fully.

### Inner Setting

For some practices, the *Physical Infrastructure* served as a perceived barrier with practices indicating a lack of available exam rooms to perform RN visits. While *Information Technology (IT) Infrastructure* had minimal discernible impact for the majority of study sites, one institution that employed the Touchworks EHR platform (vs. Epic) was unable to seamlessly integrate key components of the innovation tools used in the RN visits, such as automating the risk calculators. The innovation was seen as having *Mission Alignment*; however, its facilitating impact was negated by other barriers.

Implementation was facilitated in practices with existing RN delegation protocols by a culture of *Learning-Centeredness* where RNs and practice staff were interested in exploring how the intervention could add value to those protocols. More generally, however, most practices either did not prioritize learning new workflows and tools or lacked the bandwidth to do so.

In addition to internal communications, the ready *Access to Knowledge and Information* given by the research team served as an important facilitator for implementation; research teams provided practices with *Available Resources* in the form of online training sessions, information sheets and guides, study presentations, and frequent communication to help support use of the tools and adherence to the study workflow. While access to these resources was seen as a positive influence on implementation, some practices and personnel expressed general confusion about the study despite educational efforts on the part of the study site research teams.

### Individuals

#### Roles Subdomain

Overall, while there was buy-in among *High-Level Leaders*, the impact of their buy-in, without greater system changes, did not noticeably influence implementation. At institutions that had strong buy-in from high-level leaders, this helped to support recruitment and study implementation. Buy-in from *Mid-Level Leaders* like RN/practice managers did not have any notable impact on site success; however, resistance from mid-level leaders would have likely served as a strong barrier to implementation. Conversely, *Opinion Leaders* had a strong negative influence on implementation. While they were rare to identify, when opinion leaders had negative thoughts about the program, this had a strong impact on others involved in the intervention. For example, an RN at one practice led a petition, signed by all RNs in the practice, for the practice to not participate in the study. When that RN left the practice, however, the practice’s nurse leadership returned to the research team to express interest in participating. At another practice, providers who held unfavorable views of the study actively hindered implementation by deleting study-related notes, deterring RNs from continuing to participate.

The presence and support of *Implementation Facilitators* were a positive influence on the study as they helped research staff in identifying practices for recruitment, supporting the nursing team, and addressing study barriers. In some instances, *Implementation Leaders* and *Implementation Team Members,* including nursing leadership, facilitated implementation by allowing RNs to receive paid time for trainings, identifying RN advisory committee members, and actively supporting the research and practice staff.

Overall, the level of involvement, or lack thereof, from *Innovation Deliverers* (RNs) and *Innovation Recipients* (patients) served as barriers to implementation. At most institutions, RN participation was low due to a combination of factors discussed in other constructs. Similarly, at the majority of institutions, patients were generally not very open to the concept of an RN visit, preferring to be seen by a provider. In one example, participating urgent care practices mentioned that their patients were typically in distress and desired to be seen quickly by a provider.

### Implementation Process

*Assessing Context* and *Assessing Needs* prior to implementation were helpful for adjusting the innovation for uptake and adoption. Through meetings, interviews, and observations, research staff were able to collect extensive information about potential barriers and facilitators as well as the priorities and needs of the practice staff, especially the *Innovation Deliverers* (RNs), to guide the implementation strategy and tool changes. Despite having initial plans for implementation, however, research teams were often unable to carry out their implementation strategies to optimize delivery of the innovation tools *(Doing)* due to barriers within other constructs*.* Efforts related to *Planning* and *Tailoring Strategies* ultimately did not have any discernable impact on implementation since any positive impact was negated by barriers identified above such as COVID-19, workflow compatibility, and practice attitudes.

On the other hand, *Teaming* helped facilitate implementation as research teams worked closely with practice staff, IT teams, and data teams to coordinate and carry out different study-related tasks. Research teams also made extensive efforts to encourage practice participation, which was a driver of practice participation at most institutions. *Engaging* the *Innovation Deliverers* (RNs) had a strong positive influence on the study as research teams worked closely with the practice RNs to encourage and incentivize them to use the innovation tools.

Finally, *Reflecting and Evaluating*, as well as *Adapting* based on evaluation results, facilitated study implementation. Research teams across all institutions continuously collected feedback and data, both formally and informally, in order to adapt the *Innovation* and *Implementation*. Findings were used to identify areas for targeted intervention, make changes to training resources, and improve communication strategies. Practices at one institution were also very proactive in providing feedback and suggestions for improving the iCPR tools, many of which were shared with other study sites and implemented to improve tool usability and ensure tool uniformity.
